# Hydrotransport-Oriented Zn, Cu, and Pb Behavior Assessment and Source Identification in the River Network of a Historically Mined Area in the Hokuroku Basin, Northeast Japan

**DOI:** 10.3390/ijerph16203907

**Published:** 2019-10-15

**Authors:** Qingqing Lu, Zhengfu Bian, Noriyoshi Tsuchiya

**Affiliations:** 1 School of Environment Science and Spatial Informatics, China University of Mining and Technology, No1, Daxue Road, Xuzhou 221116, China; qqlu@cumt.edu.cn; 2 Graduate School of Environmental Studies, Tohoku University, Aoba 6-6-20 Aramaki Aoba-ku, Sendai 980-8579, Japan; tsuchiya@mail.kankyo.tohoku.ac.jp; 3 Collaborative Innovation Center for Resource Utilization and Ecological Restoration of Old Industrial Base, China University of Mining and Technology, Xuzhou 221116, China

**Keywords:** heavy metal flows, source analysis, accumulation, historically mined area

## Abstract

Aquatic ecosystems continuously receive potentially hazardous heavy metals from natural and anthropogenic sources. Focusing on the origin of heavy metals, this study aims to estimate the load contribution of tributaries from individual watershed and human drainage and to dissect the source of heavy metals, as commonly required for environmental impact assessment. Using integrated water dynamics, Geographic Information System (GIS), and chemical analysis, we identified and evaluated the heavy metal sources of the Kosaka river system in Hokuroku basin, which is a historically mined area in Northeast Japan, both in the high-water and low-water seasons. The migration and diffusion behaviors of heavy metals along with hydro-transport were analyzed, and the effects of mining activities on regional water quality both in the high-water and low-water seasons were clarified. The results indicate that Zn pollution was obvious in the Kosaka River network, especially in the downstream area. The spatial heterogeneity of heavy metal outflows from tributary watersheds was obvious, and the variations had strong correlations with mine site locations. The heavy metal flows in the mainstream increased sharply in the vicinity downstream of the Kosaka refinery drainage outlets. Compared to the low-water season, the influences of human drainage were slighter in high-water season, with lower contribution rates due to the dilution effect of the greater water discharge. Downscale sampling is effective to identify pollutant sources in regional basins.

## 1. Introduction

Heavy metal contamination in river water increases environmental risks such as toxicity [1], particularly with respect to copper, zinc, and arsenic, etc., which may be representative pollution elements of mine drainage [2,3]. Mine drainage contains several heavy metals and related high-risk elements, resulting in a variety of effects on river water quality [4,5]. Thus, the decline of water quality in rivers, lakes, and groundwater caused by these kinds of human emissions has progressively become a global issue [6,7]. Furthermore, there are naturally occurring rocks or soils with high heavy metal enrichment. As this kind of geological material reacts with rainwater or groundwater, high heavy metal accumulation will be generated in rivers. This can be regarded as naturally occurring high heavy metal accumulation [8,9,10,11]. Heavy metals introduced naturally through the weathering of land cover as well as through a variety of human activities, including mining, smelting, and agricultural practices, have released countless tons of trace elements into the environment [12,13,14,15].

Water quality reflects not only its origin but also what it encounters along its flow path. River systems that flow through mining regions are generally contaminated with metals and metalloids even long after the mining activity has ceased [16,17]. This phenomenon is due to the persistence of contamination sources including both mining drainage and the natural leaching of mining wastes, tailings, and smelting slags that remain on sites as well as contaminated sediments stored downstream in riverbeds [18]. As a point source pollutant, mining drainage directly affects regional water quality when poured into river systems. In another hand, mining districts show relatively high amounts of heavy metal concentration due to regional mineralization and/or related geological processes [19,20,21]. High heavy metal concentrations in soils and rocks in mining district have some effects on the enrichment of heavy metals in river water and ground water [22]. Both point sources and diffuse sources contribute to the total metal load of rivers, and they can be distinguished by using inventories and by using basic hydrological relationships between discharge and transported load [23].

Mining operations have significant adverse water quality impacts [24]. Most of the metal mines in Japan have already been closed; however, there still is mine wastewater being generated from the remaining deposits or mine waste residue from abandoned mines. Mining-related pollution affects not only the immediate vicinity of mines but also the hydrosystem further downstream [25]. The migration and diffusion of heavy metals in water systems and how they impact on water quality in surroundings or downstream water bodies still remain a challenge [26,27,28].

In order to evaluate environmental risks of heavy metals and arsenic caused by mining activities, the effects and relations with respect to the natural environment, artificial pollution due to mine waste residue, and emissions as a result of human activities should be estimated. Another challenging task for water management is to identify the main anthropogenic pollution sources and to assess their downstream environmental impacts [28,29]. Geographic information systems (GIS) are widely employed in the environmental studies to approximate the regional causes of water pollution [30,31,32,33] and land use/land cover effects on water quality [34,35,36].

This study integrated GIS, water dynamics, and chemical analysis together in order to: (1) identify and evaluate the heavy metal sources of river system in the Hokuroku mining area by assessing the load contributions of tributaries or human drainage from individual sub-watersheds; (2) analyze the heavy metal migration and diffusion behaviors along with water mixing through river confluence; and (3) clarify the effects of mining activities on regional water quality both in the high-water season and low-water season.

## 2. Materials and Methods

### 2.1. Site Description

**Figure 1:**
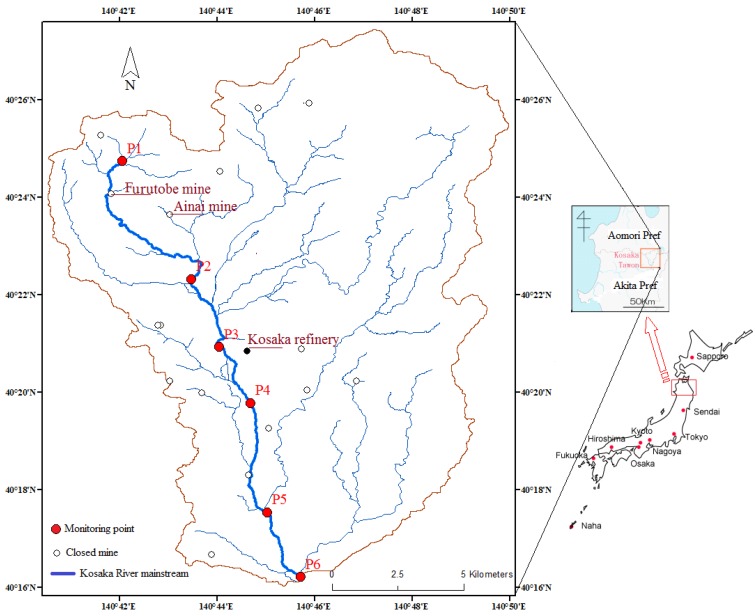
Scope of the study area: the Kosaka watershed in Hokuroku basin, Northeast Japan.

The Hokuroku basin is located in Northeast Japan (Figure 1). It is a volcano-sedimentary basin that developed within the rift graben and has been designated as the most important Kuroku ore field in Japan [37]. The total amount of the ore in this district is estimated to be approximately 140 million tons, with the average ore grade being 1.6 percent copper (Cu), 3.0 percent zinc (Zn), and 0.8 percent lead (Pb) [38,39]. It is one of the most famous and important mining districts for Zn–Pb–Cu–Au–Ag and massive sulfide deposits formed on the seafloor [40]. The Hokuroku basin is one of the most popular mining areas in Japan. The mine operated from 1916 to 1972 [41].

The Kosaka River Basin considered in this study is located in the Hokuroku Districts, with an area of about 178.5 km^2^. The mainstream river is the Kosaka River, the length of which is about 20 km. There are hundreds of years of mining history here, but all the mines are closed now. The abandoned mines are spread along the sides of the Kosaka River, and more than 10 of them produced silver, lead, zinc, copper, and other minerals in the past. At present, mine wastewater management is still occurring in the Furutobe mine and Ainai mine. The locations of the Furutobe mine, Ainai mine, and Kosaka Refinery are illustrated in Figure 1. Detailed information on wastewater discharge can be found in in Table S1. We set six monitoring points along the mainstream Kosaka River, namely P1–P6, from upstream to downstream (Figure 1). The wastewater of the Furutobe mine and Ainai mine were mixed into the Kosaka River between monitoring points P1 and P2 after proper treatment. The Kosaka refinery, as a metal recycling factory, still operates. The wastewater from the Kosaka refinery has been pouring into the mainstream river between monitoring point P4 and P5. Monitoring point P1, located in the upper reaches of the Kosaka River (the watershed of which is fully covered by forestry), is the least impacted by mines or other human activities. There are households and paddy fields downstream from P3.

The area has a sub-frigid humid climate and has four distinct seasons. It is subject to heavy monsoons and little sunshine, which results in heavy snowfall during the winter. The coldest month is January, with temperatures averaging −3 °C, whereas warmest month is August, with temperatures averaging 22 °C. The highest and lowest precipitation appear in July and February, respectively. The snow-melting period is in April and early May, and the discharge increases significantly in the rivers.

### 2.2. Watershed Division and Water Discharge Measurement

**Figure 2:**
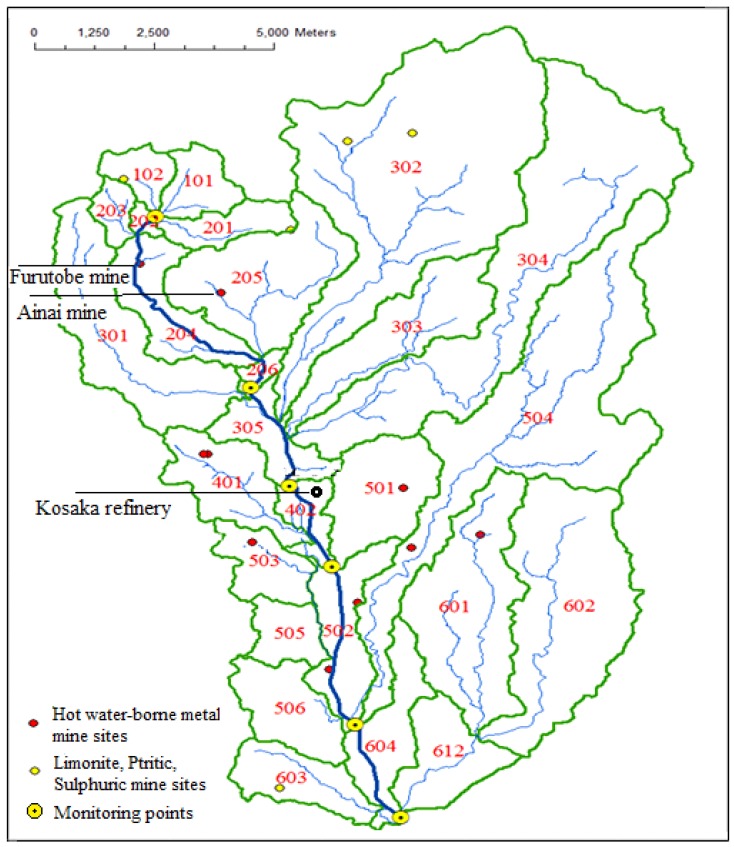
Sub-watershed division of the study area and historical mine site locations.

Ten-meter resolution Digital Elevation Model (DEM) of the study area was applied to divide the whole study area in ArcGIS 10 (Esri). This process was carried out in our previous work; the detailed methods can be found in the published paper [42]. The Kosaka River Basin was divided to 26 sub-watershed polygons. The polygon information is shown in Figure 2 and Table S2. Eighteen of these sub-watershed polygons were individual tributary polygons. Considering the complexity of mainstream polygons, the later analysis scarcely involved them.

**Figure 3:**
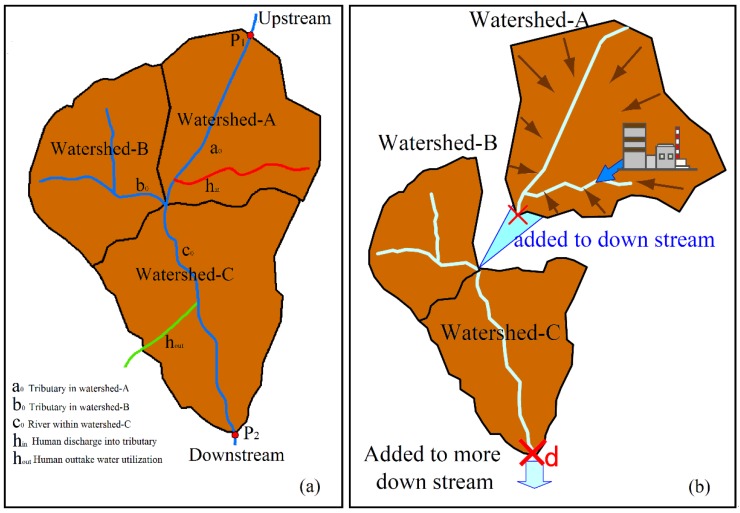
Schematic diagram of water flows and heavy metal accumulation in the river system: (**a**) Distribution of river system; (**b**) Heavy metal accumulations from upstream to downstream.

**Figure A1:**
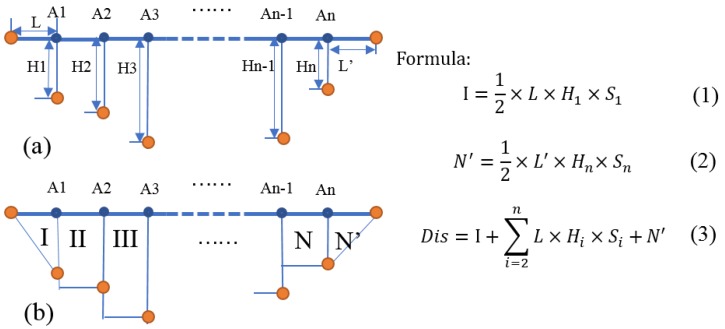
Instantaneous water discharge determination of river cross section at the monitoring point: (**a**) Sketch map for the division of river section; (**b**) Approximate calculation of river section. Starting from river bank, measuring points were set at intervals of *L(m)*, *A_i_* is the measuring points; The depth and flow velocity of the river water was measured at each measuring point, namely *H_i_ (m)* and *S_i_ (m/s)*; I, II, III, …., N, N+1 *(m^3^/s)* are the instantaneous water discharges of the divided cross sections including two triangles and a set of trapezoids, and the sum of which was taken as the instantaneous water discharge of the river cross section at the monitoring point.

For accurate heavy metal mass flow estimation, heavy metal concentration and water discharge values should optimally be measured simultaneously over a time period and from the same sampling point. In this study, the water discharge at the six monitoring points along the mainstream Kosaka River were actually measured by multiplying the river cross section and the average water flow velocity, both in the high-water season (May 2015) and the low-water season (October 2015). Water flow velocity was measured using a current meter, while the cross section was approximated to a set of trapezoids, and the sum was taken as the cross section. The specific calculating methods are given in Appendix A Figure A1. Water discharge measurement of all the tributaries requires a great deal of work [43]. Due to time and cost constraints, it is difficult to accomplish this work and this is a key problem in the field of hydrology. With the addition of the inconvenience field operation in the study area, the field measurements of tributary water discharge have not been carried out. Furthermore, the tributaries are located in individual sub-watershed polygons mostly derived from natural precipitation, and have a slight anthropogenic influence. Only from the consideration of natural gaining water is the water discharge of the rivers considered to be directly proportional to the river basin area. Thus, the water discharge of each tributary polygon was calculated by the weighted averaging of interzone water increase between two monitoring points in this study according to the formula

(1) $$Q={\mathrm{Dis}}_{\left(i\right)}\times \left(\frac{A}{A+B+C}\right)$$

where *Q* is the water discharge of watershed *A* (Tributary a_0_); ${\mathrm{Dis}}_{\left(i\right)}$ is the natural increase of interzone water discharge between monitoring point P1 and P2; and *A*, *B*, and *C* are the areas of the sub-watershed polygons, respectively (Figure 3a).

The water discharge at monitoring points depends on both the natural precipitation and the anthropogenic influence. It is necessary to deduct the part caused by human activity from the actually measured water discharge between each two monitoring points, so as to acquire an accurate estimate of the relationship between the watershed area and the precipitation. The natural precipitation part of water discharge between two monitoring points can be calculated using the following formula:

(2) $${\mathrm{Dis}}_{\left(i\right)}={\mathrm{Dis}}_{\left(d\right)}-{\mathrm{Dis}}_{\left(u\right)}-{\mathrm{Dis}}_{\left(h\right)}$$

where ${\mathrm{Dis}}_{\left(d\right)}$ is the discharge at the downstream monitoring point (P2 in Figure 3a); ${\mathrm{Dis}}_{\left(u\right)}$ is the water discharge at the upstream monitoring point (P1 in Figure 3a); and ${\mathrm{Dis}}_{\left(h\right)}$ is the sum of human discharge between the two monitoring points. ${\mathrm{Dis}}_{\left(i\right)}$ is the interzone increase of water discharge between the two monitoring points, and corresponds to the natural precipitation part. Similarly, in order to display the natural precipitation part of the water discharge at the monitoring points, the human water intake and drainage in the upstream area were deducted, obtaining the revised water discharge values.

### 2.3. Sampling and Experiments

Water sampling was conducted at the six monitoring points (Figure 1) along the mainstream Kosaka River both in the high-water season (April 2011, April 2014, and May 2015) and low-water season (November 2012, October 2013, and October 2015). Furthermore, 19 water samples were collected from each tributary ahead of the outlet where they flow into the mainstream both in the high-water season (May 2015) and low-water season (October 2015).

Water samples were sampled from 0.5 meters below water surface. All water samples for dissolved Zn, Cu, Pb and As analysis were filtered on-site (through a 0.2 μm pore membrane) to separate out the larger particles, and then acidified (HNO_3_) to prevent the metal ion hydrolysis precipitation prior to analysis. Zn, Cu, Pb, and As were selected for source analysis because of their association with mining activities. The Zn, Cu, Pb and As contents were analyzed by ICP/MS (Perkin Elmer Sciex, Wellesley, MA, USA). In this study, all the experiments were carried out in an ultra-clean environment. Milli-Q water was used for all experiment water use. The reagents used in the experiment were high-purity reagents [44]. The experimental operation was undertaken strictly as per Japan Industrial Standards [45]. We ran a group of blanks every 20 samples while operating the ICP-MS test to conduct the quality control in the analysis and test process through the blank deduction. The Standard Reference Material (SRM 1643e) was brought into the analytical test after blanks [46]. The accuracy of the experimental results is analyzed by the deviation between the actual measured value of the standard substance, and the relative standard deviation (RSD) was less than 5%.

Mine wastewater drainage still continues in the Furutobe mine, Ainai mine, and Kosaka refinery. Data and information on these anthropogenic emissions are obtained from municipality and autonomous bodies in Kosaka, Akita Prefecture, including the monthly discharge information of the Furutobe mine and Ainai mine. The yearly average discharge information on the Kosaka refinery was obtained from the Pollutant Release and Transfer Register (PRTR) of the same. The specific information on these anthropogenic emissions can be found in Table S1.

Hierarchical cluster analysis were carried out in SPSS version 21.0 to group the individual tributaries. The specific information on this statistical analysis can be found in other works [47,48].

### 2.4. Theoretical Considerations

Consistent with other catchment-scale models, an individual sub-watershed polygon can be regarded as a conceptual model of a non-point contaminant source zone. The water gathering of the tributary outlet to the mainstream is fed by small, non-permanent streams and/or diffuse flows of ground water, which are fully located within this sub-watershed polygon. Therefore, the contaminant mass cannot be lost to other tributaries out of this polygon. That is, for an individual non-point source, the contaminant outflow at the outlet to the mainstream depends on and only depends on all the internal features of the sub-watershed polygon. The heavy metal mass outflow depends on the local concentration and the discharge, the model of which is as follows:

(3) $$Mf=C_{i}\times Q_{i}$$

where *Mf* is the heavy metal outflow of an individual watershed polygon; *C_i_* is the heavy metal concentration of a tributary “*i*”; and *Q_i_* is the water discharge through the outlet of that tributary polygon.

The dissolved heavy metal flows and accumulates from upstream to downstream as the river water flows, and the heavy metal content together with the flow rate changes at junctions. The water flow rate and the heavy metal content at the given point on the mainstream can be determined as the sum of those from all polygons upstream (Figure 3b). The water and heavy metal flows mix together at confluences. The confluence effects of the upstream discharges can be expressed by the following formula:

(4) $$Mf_{conf}=\underset{i}{\sum }C_{i}\times Q_{i}$$

where *Mf_conf_* is the heavy metal outflow at the downstream of the confluence; and “*i*” refers to the tributaries or human discharges at the upstream of the confluence. “*C*” and “*Q*” are the heavy metal concentration and water discharge of the tributaries or human discharge.

## 3. Results

### 3.1. Water Discharge

**Figure 4:**
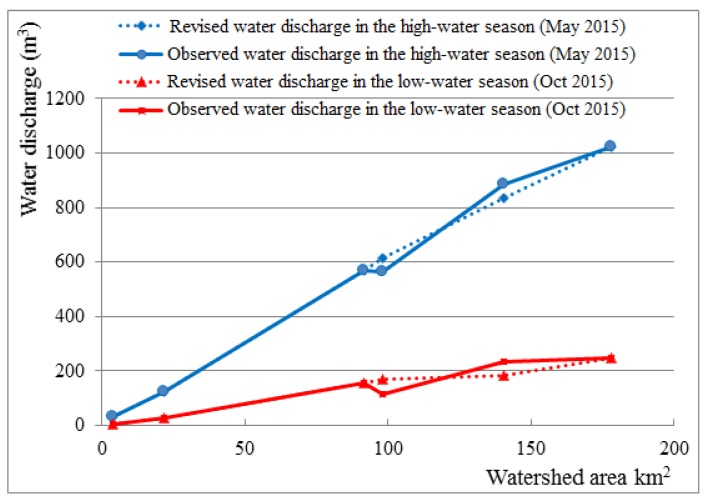
Water discharge at the monitoring points both in the high-water and low-water seasons.

Figure 4 shows the water discharge at the monitoring points both in the high-water season and low-water season. The solid lines show the actually measured water discharge, while the dotted lines stand for the revised values which deducted the human influence. The water discharge in the high-water seasons was relatively large, since the snow-melting season in the study area is from late March to early May. The water discharge at monitoring point P6 in the high-water season was roughly five times the amount of that in the low-water season. The revised water discharge values were mostly higher than the actually measured ones. The difference was regarded as the result of human activities. Figure 4 is plotted by the water discharge values at the monitoring points corresponding to their watershed areas, revealing relationship between the water discharge and the watershed area both before and after the revision of human effects. Generally, the water discharge generated by natural precipitation would be linearly correlated to the corresponding watershed areal. In Figure 4, the shapes of dotted lines are closer to straight lines. To some extent, this reveals that the revised water discharges were more natural, showing the advisability of water discharge measurements in this study.

Water discharge values from each tributary polygon were calculated based on the actually measured water discharges at the monitoring points both in high-water season and low-water season. The results can be found in Tables S1 and S6. These water discharge data of tributary polygons will be employed in the mass balance analysis of Zn, Cu, Pb and As flows in the latter part of this study.

### 3.2. Zn, Cu, Pb and As Behaviors Mainstream

**Figure 5:**
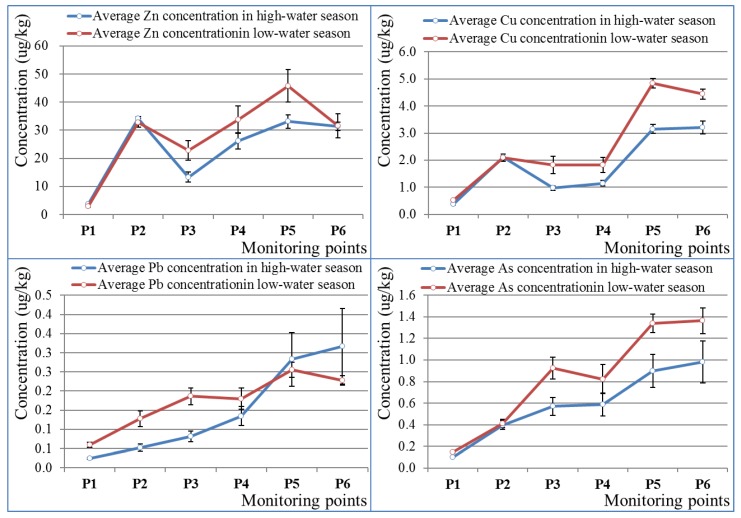
Zn, Cu, Pb and As concentrations along the mainstream Kosaka River both in the high-water season and low-water season. The error bar is plotted by the standard error (SE) of the Zn, Cu, Pb and As concentrations in the high-water season (April 2011, April 2014, and May 2015) and low-water season (November 2012, October 2013, and October 2015).

The average values of Zn, Cu, Pb and As concentrations for the three periods at the monitoring points were calculated in the high-water season and low-water season, respectively (Figure 5). The blue lines in Figure 5 stand for the average Zn, Cu, Pb and As concentrations in the high-water season at the monitoring points, which were calculated from the data from April 2011, April 2014, and May 2015. Similarly, the red lines indicate the average Zn, Cu, Pb and As concentrations in the low-water season at the monitoring points, calculated from data from November 2012, October 2013, and October 2015. The results shown in Figure 5 show that both in high-water season and low-water season the concentrations of Pb and As were lower than 10 ug/L (Environmental Quality Standards of Human Items, Japan) mainstream [49], while the concentrations of Cu were lower 5 μg/kg (Environmental Quality Standards for Specific Pollutants, EU) [50]. Meanwhile, Zn in the mainstream Kosaka River obviously exceeded the standard of 30 μg/kg (environmental quality standards of living environment items, Japan) [49,51], especially in the downstream area. Both in high-water season and low-water season the Zn concentrations at monitoring points P2, P5, and P6 were beyond the standard, and Zn concentrations at monitoring point P4 were also beyond 30 μg/kg in the low-water season.

From upstream to downstream, Zn and Cu concentrations increased between P1 and P2 and decreased between P2 and P3, and then increased again between P3 and P5 afterwards decrease again. There were two peaks, at P2 and P5, respectively. There are human drainage areas located before monitoring points P2 and P5. The concentrations of both As and Pb gradually increased upstream to downstream. The variation trend of these two elements is quite different from those of Zn and Cu. Thus, human emissions did not generate points of element concentrations on account of the low concentrations of Pb and As in human drainage. The increasing concentration of As and Pb in downstream might influenced by the increasing external disturbance, since the upstream watershed is more natural as it is covered with forest.

In terms of seasonal variation, Zn and Cu concentrations in the low-water season were relatively larger than those in the high-water season in the downstream area, while there were almost no obvious differences in Zn and Cu concentrations between the high-water season and the low season at P1 and P2. The upstream watershed of P2 was more natural than in the other section of the whole Kosaka watershed area since there is no effective human activity (it is completely covered by forest, with no paddy fields). Zn and Cu concentrations of downstream monitoring points were influenced by both the Kosaka refinery drainage and human activities (paddy fields). The phenomenon where Zn and Cu concentrations were lower in the high-water season was especially clear downstream. The larger water flow rate in the high-water season can effectively dilute the pollutants. Therefore, reducing the mine drainage in the low-water season should be proposed. As concentrations in the low-water season are larger than in the high-water season, and the differences between them do not vary greatly in the downstream area. The same situation occurs for Pb upstream of monitoring point P4. Pb concentrations downstream in the high-water season exceeded the values of the low-water season. This may be caused by the extremely large seasonal variations at monitoring points P5 and P6.

### 3.3. Zn, Cu, Pb and As Behaviors in Tributaries

**Figure 6:**
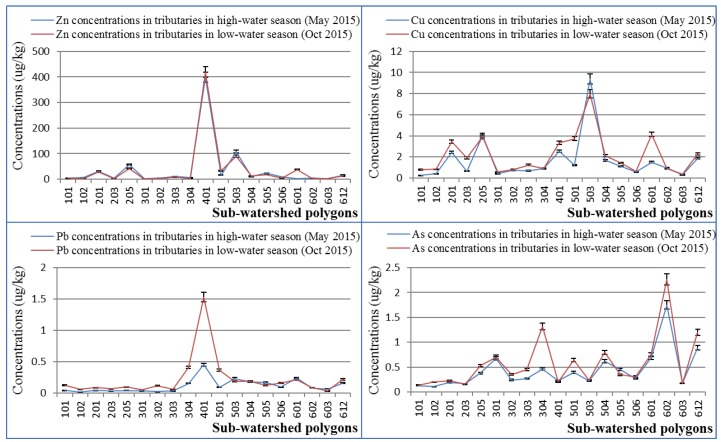
Zn, Cu, Pb and As concentrations in tributaries both in the high-water and low-water seasons. The error bar was plotted following the maximum allowable relative standard deviation of 5%, referring to the precision of the Standard Reference Material (SRM 1643e) analysis.

The concentrations of Zn, Cu, Pb and As in tributaries both in the high-water season and low-water season are shown in Figure 6. There were remarkable spatial variations of the concentrations of Zn, Cu, Pb and As among different sub-watershed polygons. The concentrations of Zn, Cu, Pb and As were larger in the low-water season compared to the high-water season by and large.

In the majority of the sub-watershed polygons the Zn concentrations were above those stipulated by the Environmental Quality Standard for Surface Water in Japan (30 μg/kg). The contents of Pb and As in river water in the study area were generally below those stipulated by the Environmental Quality Standards of Human Items in Japan (10 μg/kg). Zn pollution was serious in tributaries at the watershed scale in the study area, especially in some parts like the polygon 401 area. The Zn concentration in polygon 401 reached up to about 419 μg/kg in low-water season and up to about 398 μg/kg in high-water season, values above the standard. These Zn-polluted tributaries flowed into the mainstream Kosaka River, contributing to high Zn concentrations mainstream. In addition, there also was an obvious peak in polygon 401, which had the largest Pb concentration. The concentrations of Zn, Cu, Pb and As varied greatly among different tributaries; however, the variation characteristics of them were different. The largest Cu concentration occurred in polygon 503 both in the high-water season and low-water season, respectively corresponding to 7.96 μg/kg and 9.37 μg/kg. For As, polygon 602 showed the largest concentrations both in the high-water season and low-water season. Therefore, polygon 401 has an obvious Zn–Pb signature, and could be named the Zn–Pb polygon. Similarly, polygon 503 and polygon 602 were named the Cu polygon and As polygon, respectively.

### 3.4. Zn, Cu, Pb and As Outflows into the Mainstream Kosaka 

**Figure 7:**
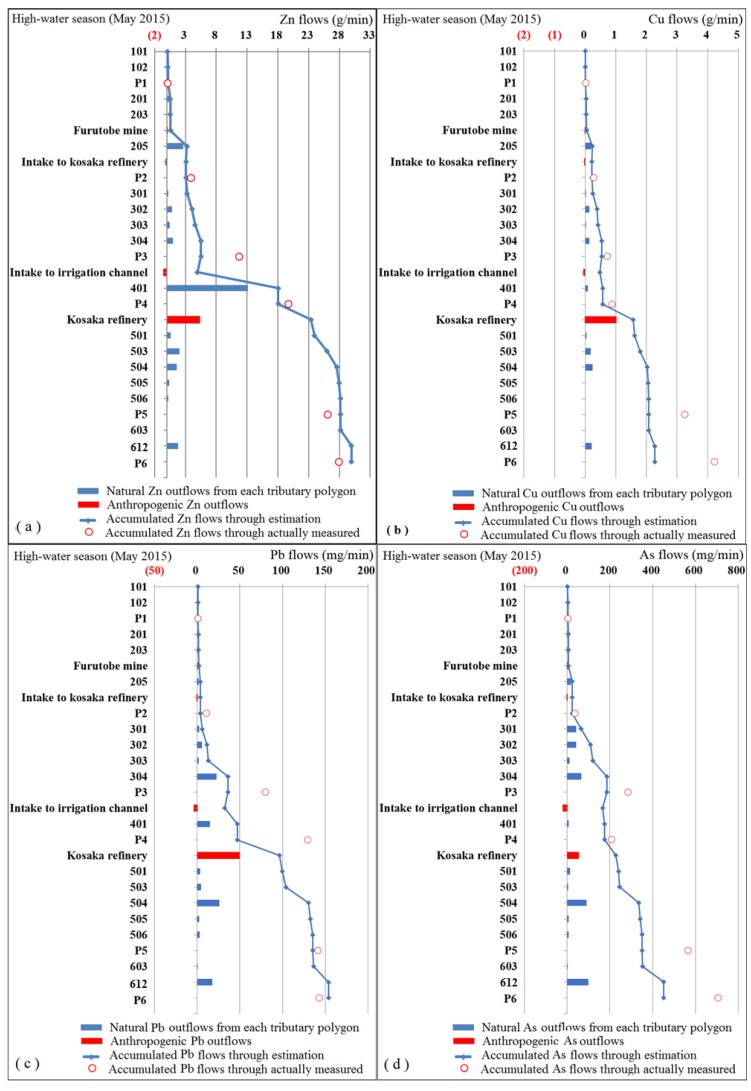

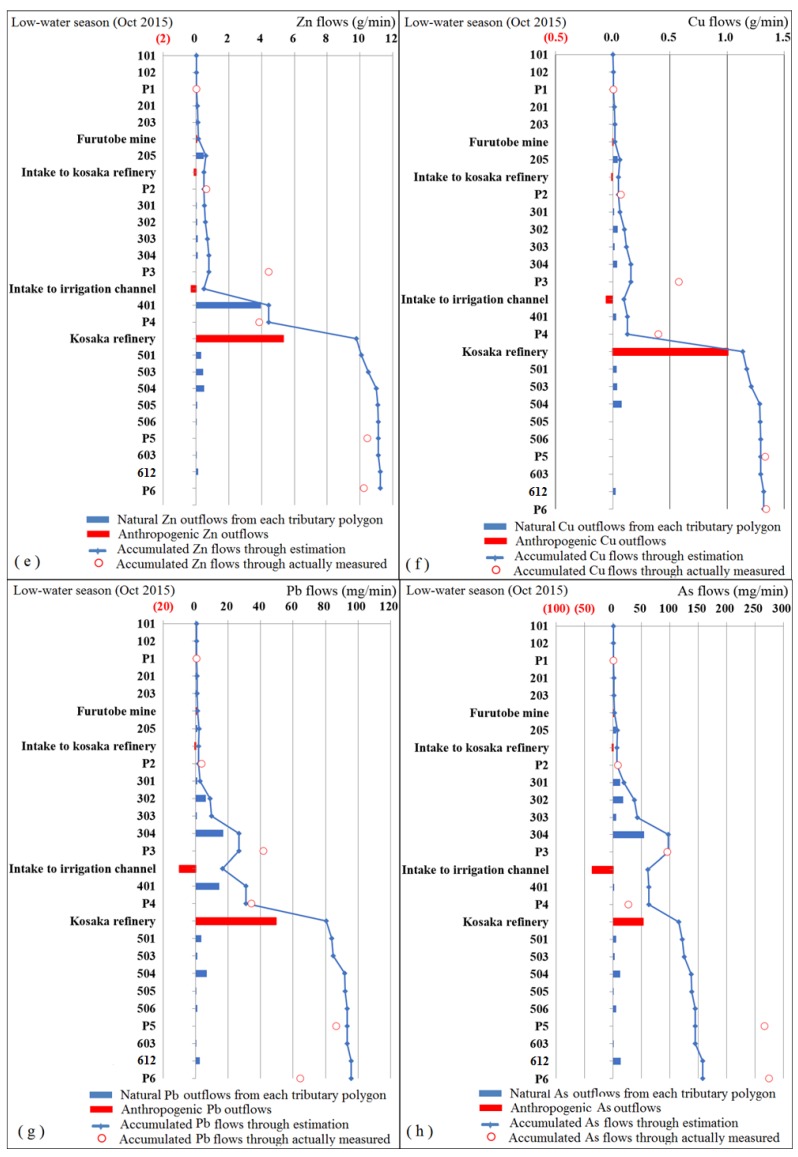
Zn, Cu, Pb and As outflows or inflows to the mainstream Kosaka River both in the high-water and low-water seasons: (**a**) Zn flows in the high-water season; (**b**) Cu flows in the high-water season; (**c**) Pb flows in the high-water season; (**d**) As flows in the high-water season; (**e**) Zn flows in the low-water season; (**f**) Cu flows in the low-water season; (**g**) Pb flows in the low-water season; (**h**) As flows in the low-water season. The red numbers in parenthesis are negative, standing for the outflows from the mainstream. The blue bars stand for Zn, Cu, Pb and As outflows of the non-point source part, corresponding to the natural outflows from each tributary polygon. The red bars stand for the point sources, expressing the outflows caused by human activities. The blue lines stand for the accumulated Zn, Cu, Pb and As flows of the upstream watershed. The small red circles stand for the observed Zn, Cu, Pb and As flows at the monitoring points.

Soluble heavy metals in river water are transferred with water flows into the watershed. The unit times of heavy metal outflows into the mainstream from each tributary polygon and human drainage point were calculated, multiplying the water discharge and heavy metal concentrations of tributary drainage and human drainage points into the mainstream river. The data sets of soluble Zn, Cu, Pb and As concentrations (filtered by 0.2 μm pore membrane) and water discharge in May 2015 and October 2015, which corresponded to the high-water season and low-water season, respectively, were performed. The calculation results for Zn, Cu, Pb and As can be found in Supplementary Tables (Tables S1, S3–S9). In order to visually display the spatial differences and seasonal changes of Zn, Cu, Pb and As outflows in tributary polygons, Figure 7 was plotted.

In Figure 7, the blue bars stand for the Zn, Cu, Pb and As outflows of non-point source part, corresponding to the natural Zn, Cu, Pb and As outflows from each tributary polygon. This part is derived from natural surface runoff that carries the heavy metals naturally dissolved out from land cover. The rainwater flows through and acts with the surface soils or rocks within the watershed before reaching the rivers. It was found that the blue bars change a lot between different polygons, revealing the spatial heterogeneity of Zn, Cu, Pb and As load contribution to the mainstream, and had different influences on mainstream water quality. The red bars stand for the point sources, expressing the outflows caused by human activities, showing that dominant human drainage significantly affects the local river water quality, especially for Zn and Cu. The contribution rate of Zn, Cu, Pb and As discharge from the Kosaka refinery was relatively high compared to the natural outflows from tributaries, especially in low-water season. The natural Zn, Cu, Pb and As outflows from tributaries in the high-water season were approximately twice those in the low-water season, for all the elements involved. These increases were mainly caused by the contribution of natural precipitation, since the human drainage involved fixed amounts that did not change with the seasons.

## 4. Discussions

### 4.1. Mass Balance Analysis

Theoretically, it is possible to estimate heavy metal content downstream based on the upstream discharge information by mass balance analysis [4,52,53]. Similarly, it is also possible to estimate the heavy metal content in an unmonitored tributary branch when knowing the right upstream and downstream heavy metal discharge information [4,42,54]. In this study, mass balance analysis of Zn, Cu, Pb and As confluence from upstream to downstream was carried out both in the high-water and low-water seasons. The estimation of Zn, Cu, Pb and As loads mainstream, representing the accumulated flows of the upstream watershed, are shown in blue lines in Figure 7. We applied the observed Zn, Cu, Pb and As loads at monitoring points to examine the accuracy of the estimations, which were separately obtained by multiplying the actual measured water discharge and the concentration data analyzed by ICP-MS. On the whole, the small red circles in Figure 7, which represent the observed Zn, Cu, Pb and As loads at the monitoring points, were in agreement with the blue lines. However, some obvious deviations were shown in some of the monitoring points. The specific relative errors for the estimation of Zn, Cu, Pb and As loads mainstream based on accumulative effects of the upstream outflows are shown in Table 1. For different monitoring points, the absolute values of average relative errors ranged from 6.63% to 44.76%. With an exception at monitoring point P1, all others showed a negative deviation. There were continuous underestimations of the downstream loads of Zn, Cu, Pb and As. The discrepancies are partly due to not including the polygons along the mainstream, since these mainstream polygons account for about 11.14% of the whole study area. There would be considerable actual discharge that has not been accounted for in the model. In the mainstream polygon, there was afflux from circumjacent tributaries in addition to the surface runoff collection within the polygon. Its complexity makes it difficult to be considered in the estimation model in this study. Another reason for the large discrepancies might come from the technical level in terms of the measurement accuracy of concentrations and water discharge. Relatively speaking, the estimation at monitoring point P1 absolutely conformed to the model since there was no mainstream polygon and hydrological conditions are relatively simple. The average relative error of 9.6% could authentically reflect the model accuracy, proving that the metal loadings could reasonably be modeled by mass balance analysis. Josefin et al. also proved this theoretical consideration [4,43,53]. However, theoretically feasible models are not applicable to all situations. For large-scale watersheds, the complex confluences of tributaries and human discharges or water intaking would increase the difficulty of estimation but also decrease its accuracy. The estimation accuracy shown downstream was less than satisfactory (Table 1).

Nevertheless, the mass balance analysis did a good job of explaining the sources of Zn, Cu, Pb and As mainstream, specifying where they came from and where they went as well as their contribution to mainstream load increase or decrease. The accumulated Zn, Cu, Pb and As flows gradually increased from upstream to downstream. There was a suddenly sharp increase in the downstream of polygon 401 for Zn, caused by the extremely large contribution rate of Zn flow added by polygon 401. However, the accumulated Cu flows sharply increased from the downstream of monitoring point P4, mainly caused the discharge from Kosaka refinery. For Zn, Cu, Pb and As, the accumulated flows in the upstream area before monitoring point P2 contributed a relatively small part of the flow to the mainstream in the whole study area. It is concluded that the environmental effect of heavy metals in the upstream area was slight for the Kosaka watershed compared to the downstream area, sharply increasing due to the large heavy metal flows from the tributary polygons and Kosaka refinery. Compared to the high-water season, the effect of human drainage on mainstream water quality in the low-water season was more remarkable, with a larger contribution rate than natural outflows from tributaries. The influences of human drainage were slighter in the high-water season, with a lower contribution rate due to the dilution effect of the greater water discharge. This is consistent with the results of Gozzard et al., who reported that sub-watershed nonpoint sources were of little importance in the low-water season, with point source human water discharge dominating instream concentration and load [55]. This is an effective way to arrange industrial water discharge during the precipitation period, reducing the effect on the aquatic environment since the large water quantity can effectively dilute the pollutants [56].

### 4.2. Source Analysis of Zn, Cu, Pb and As Pollution

There were significant spatial variations for both the concentrations and outflows of Zn, Cu, Pb and As among tributaries. Polygon 401, with an obvious Zn–Pb signature, provided considerable Zn and Pb outflows (Figure 7) in the case of relatively small water discharge due to the ultrahigh concentrations (Figure 6). It was an obvious local pollution source zone. In addition to the Furutobe mine, Ainai mine, and the Kosaka refinery, the untreated Horinai mine site in polygon 401 might be a critical pollution source to be taken into consideration. The Horinai mine site in this polygon was a vein-type deposit, where Zn dominated [39,40]. The Cu flows from Cu-polygon 503 was not large enough to affect the mainstream water quality. The slightly elevated Cu concentration, together with the minor water discharge, cannot change the overall level. The Nagaki mine in polygon 503 was a Koroku-type deposit with abundant Cu [38,41], explaining the elevated Cu concentration here. The high concentration peaks have a relationship with the mine site location, since mine sites can be found in peak polygons such as polygon 205, polygon 401, polygon 503, and polygon 601 (Figure 2, Figure 6). We considered that the peaks of Zn and Cu concentrations at P2 (Figure 5) were most certainly caused by Furutobe mine drainage and the Ainai mine in polygon 205 located before monitoring point P2, because there were no other sources upstream. Besides, the peaks of Zn and Cu concentrations at monitoring point P5 (Figure 5) were mainly influenced by the Kosaka refinery drainage which fluxes into the mainstream between P4 and P5 on account of the large contribution rate to the mainstream water quality (Figure 7). In conclusion, mining has a profound impact on water quality in the study area, whether from current mine drainage or non-point sources with abandoned mine sites.

**Figure 8:**
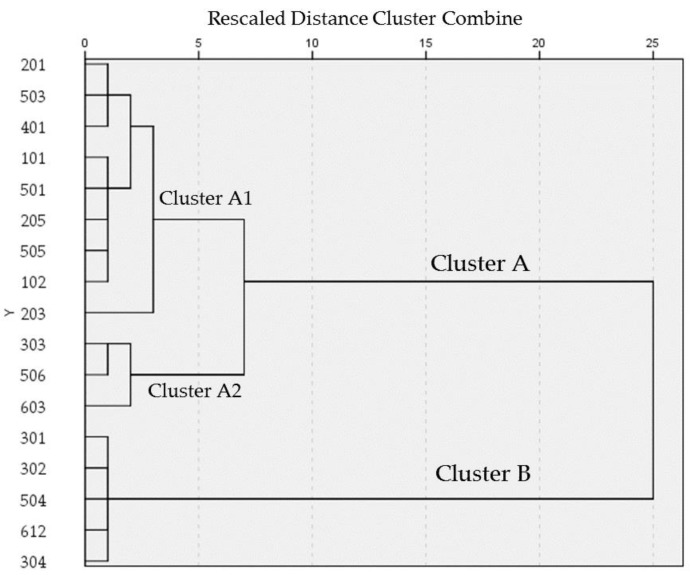
Dendrogram for hierarchical cluster analysis of Zn, Cu, Pb and As concentrations and flows (Euclidean, Ward method).

The individual tributaries were grouped into two clusters (Figure 8). Considering Zn, Cu, Pb and As concentrations and outflows of all the individual tributaries, we cannot definitively diagnose the similarities of tributaries in Cluster A. The tributaries in Cluster A2 had a common characteristic: relatively low concentrations as well as relatively small outflows of Zn, Cu, Pb and As. The tributaries in Cluster A1 presented small outflows of Zn, Cu, Pb and As overall, with the exception of Zn outflows in polygon 401. However, their concentrations presented large variations. One common characteristic of the tributaries in Cluster B is that they did not have high concentrations but did have large outflows of Zn, Cu, Pb and As. Another interesting common characteristic is that the outflows of Pb and As were considerably larger in these tributaries compared to Zn and Cu and compared to other tributaries. There was larger area of paddy field in these Cluster B tributaries (Table S2). Pb- and As-contaminated paddy fields exist in Japan [57]. The use of fertilizers, insecticides, chemical herbicides, and sewage sludge is one of the sources of Pb contamination in paddy field [57,58,59]. Pb and As contamination in paddy fields can be caused by mining, since slag in open fields containing a high concentration of Pb and As can easily flow into paddy fields and accumulate for geographical reasons [57,60,61]. Besides, converting a fruit orchard to a paddy field would also lead to As contamination [57]. As a historical mine area, the paddy fields here in all probability have Pb and As contamination. That explains the larger quantity of Pb and As outflows from these Cluster B tributaries. In conclusion, the paddy in the study area was probably another significant pollution source besides human drainage and the mine site.

### 4.3. Advantages of the Integration of Water Dynamics, GIS, and Chemical Analysis Together for Environmental Investigation

As a tool, GIS can play a good role when integrated with other research techniques. GIS integrated with enrichment factor (EF) determination and nonmetric multidimensional scaling (NMS) was firstly developed to identify anthropogenic heavy metal sources in marine sediments in Hong Kong [62]. GIS-based analysis in environmental investigation plays an important role for identifying pollution sources, locating pollution sources, and assessing the spatial heterogeneity of environmental factors and their influences [62,63,64]. In this study, the main role of GIS was to provide a basic platform, helping to determine the tributary network system and sampling points and dividing the study area into several spots leading to the explication of spatial heterogeneity. Introducing the idea of water dynamics to heavy metal migration in river water is conducive to describing the heavy metal behaviors in an aquatic system since running water is the carrier [4,65]. Chemical analysis offers traditional experimental techniques for heavy metal pollution analysis. The advantages of the integration of water dynamics, GIS, and chemical analysis in this study were reflected in several aspects. Firstly, the implementation of downscale sampling downscales the large watershed area into smaller ones that effectively identifies pollution sources. Secondly, clarifying the heavy metal load contribution of each individual sub-watershed or human drainage point dissects the sources of the heavy metals and locates the specific pollution sources. Besides, an approach was provided to make a transition from the macroscopic to microcosmic level, which is helpful to monitor and control pollutant sources in regional basin. The heavy metal outflows in each sub-watershed polygon clarified the internal specific details of the pollution level of the whole watershed area, which might be ignored in large-scale pollution source investigations. The low-order stream sampling at a small scale can provide advantages for the identification of pollution sources in the tributary, compared to the more common practice of sampling larger water bodies [51,66]. Sampling from second-level branches provides higher resolution information on water quality compared to sampling from a larger-scale water body. However, the increased capacity for pollution source identification comes at the expense of more heavy work. In this study, using Zn concentrations in the mainstream Kosaka River (Figure 5) we do not imagine that a critical Zn pollution source zone (polygon 401) with a hazardous Zn concentration greater than 400 μg/kg exists in the tributary. However, with the output of this tributary into the mainstream river, high concentrations would be diluted. Meanwhile, Zn, which poses a serious threat to the microorganisms within sub-watershed 401, was well hidden in the mainstream river, evidenced by a decreased Zn concentration of about 35 μg/kg at monitoring point P4 (Figure 5). Therefore, downscale sampling is effective to identify pollutant sources in the regional basin.

## 5. Conclusions

This research investigated the Zn, Cu, Pb and As behaviors in the Kosaka watershed of a historically mined area. The motivation was to identify and evaluate the sources of Zn, Cu, Pb and As in the Kosaka River system, and further to understand heavy metal migration and diffusion behaviors. Another objective was to clarify the effects of mining activities on regional water quality both in the high-water season and low-water season. Zn pollution was severe in some tributaries, and these Zn-polluted tributaries flowed into the mainstream Kosaka River, contributing to high mainstream Zn concentrations. The study shows that Zn pollution was obvious in the mainstream Kosaka watershed, especially in the downstream area. Cu concentrations exceeding the standard are also present in some tributaries. In addition to the Furutobe mine, Ainai mine, and the Kosaka refinery, the untreated Horinai mine site in polygon 401 might be an critical pollution source which should be taken into account. The spatial heterogeneity of Zn, Cu, Pb and As outflows from the individual tributary watersheds was obvious, and the variation has strong correlations with mine site locations. The Zn, Cu, Pb and As flows in the mainstream increased sharply in the vicinity downstream of the Kosaka refinery drainage outlets. Compared to the low-water season, the influences of human drainage were slighter in the high-water season, with a lower contribution rate due to the dilution effect of the larger water discharge. Theoretically, it is possible to estimate the heavy metal flow downstream of the mainstream river by the accumulation of all the upstream tributaries and human drainage. The estimation model has a certain guarantee of accuracy for simple confluence estimation, while the estimation accuracy is not ideal for the frequent intersection of complex water systems in large-scale watersheds.

## Figures and Tables

**Table 1:** Descriptive statistics of relative errors for the estimation of Zn, Cu, Pb and As loads in mainstream.

	L-Zn	L-Cu	L-Pb	L-As	H-Zn	H-Cu	H-Pb	H-As	Average	SD
P1	−1.79%	8.43%	44.51%	17.66%	6.76%	−34.94%	25.46%	10.69%	9.60%	0.21
P2	−22.45%	−28.18%	−52.62%	−24.28%	−21.15%	−20.42%	−67.81%	−38.25%	−34.39%	0.16
P3	−82.11%	−72.95%	−36.40%	1.80%	−53.11%	−25.80%	−54.93%	−34.61%	−44.76%	0.25
P4	15.16%	−67.55%	−9.92%	128.20%	−8.47%	−35.18%	−63.66%	−16.13%	−7.19%	0.58
P5	6.22%	−2.72%	7.92%	−45.76%	7.71%	−36.45%	−4.20%	−38.17%	−13.18%	0.21
P6	9.98%	−1.61%	48.71%	−42.56%	7.28%	−46.13%	7.44%	−36.18%	−6.63%	0.31
Avg.	−12.50%	−27.43%	0.36%	5.84%	−10.16%	−33.15%	−26.28%	−25.44%	−16.10%	0.13
SD	0.33	0.32	0.38	0.59	0.22	0.08	0.37	0.18		

Notes. L-Zn, L-Cu, L-Pb and L-As respectively represent the relative errors of estimation for Zn, Cu, Pb and As loads in the low-water season. In accordance, H-Zn, H-Cu, H-Pb and H-As respectively represent these values in the high-water season.

## Supplementary Materials

The following are available online at https://www.mdpi.com/1660-4601/16/20/3907/s1, Table S1: Calculations about Zn flows into mainstream and the accumulation effects in high-water season (May 2015); Table S2. Polygons’ information; Table S3. Calculations about Cu flows into mainstream and the accumulation effects in high-water season (May 2015); Table S4. Calculations about Pb flows into mainstream and the accumulation effects in high-water season (May 2015); Table S5. Calculations about As flows into mainstream and the accumulation effects in high-water season (May 2015); Table S6. Calculations about Zn flows into mainstream and the accumulation effects in low-water season (Oct 2015); Table S7. Calculations about Cu flows into mainstream and the accumulation effects in low-water season (Oct 2015); Table S8. Calculations about Pb flows into mainstream and the accumulation effects in low-water season (Oct 2015); Table S9. Calculations about As flows into mainstream and the accumulation effects in low-water season (Oct 2015).
