# Supplementary material for: Hydrotransport-Oriented Zn, Cu, and Pb Behavior Assessment and Source Identification in the River Network of a Historically Mined Area in the Hokuroku Basin, Northeast Japan
Source: Int J Environ Res Public Health. 2019 Oct 15;16(20):3907. doi: 10.3390/ijerph16203907 (PMC6843294; doi:10.3390/ijerph16203907)
Supplement: Supplementary file 1 [file ijerph-16-03907-s001.pdf]

Table S1. Calculations about Zn flows into mainstream and the accumulation effects in high-water season (May 2015).

	AnthropogEnic Water Flows (m^3/min)	Tributary Water Discharges (m^3/min)	Zn Concentration Analyzed by ICP-MS (μg/kg)	Zn Concentration in Human Emissions (μg/kg)	Heavy Metal Outflows (g/min)	Accumulated Water Discharge (m^3/min)	Accumulated Heavy Metal Flows (g/min)	Estimated Zn Concentration in Mainstream (μg/kg)	Actually Measured Water Discharge (m^3/min)	Observed Heavy Metal Flows at Monitoring Points (g/min)
	A	B	C	D	E	F	G	H	I	J
101		18.04	4.86		0.088	18.04	0.088	4.860		
102		12.64	5.89		0.074	30.68	0.162	5.284		
P1			4.95			30.68	0.162	5.284	30.68	0.152
201		11.68	30.42		0.355	42.36	0.517	12.216		
203		6.18	3.79		0.023	48.54	0.539	11.095		
Furutobe Mine	0.23			91.741	0.021	48.77	0.560	11.475		
205		46.67	57.12		2.666	95.45	3.226	33.796		
Intake to Kosaka Refinery	−4.00 [a]				−0.135 [a]	91.45	3.090	33.796		
P2			32.34			91.45	3.090	33.796	121.20	3.920
301		62.37	3.06		0.191	153.81	3.281	21.333		
302		179.04	4.41		0.790	332.86	4.071	12.230		
303		45.21	10.47		0.473	378.07	4.544	12.020		
304		142.09	6.87		0.976	520.15	5.520	10.613		
P3			20.68			520.15	5.520	10.613	569.32	11.774
Intake to Irrigation Channel	−53.50 [a]				−0.568 [a]	466.65	4.953	10.613		
401		32.87	398.34		13.093	499.52	18.046	36.126		
P4			35.05			499.52	18.046	36.126	562.50	19.716
Kosaka Refinery	21.00			253.678	5.327	520.52	23.373	44.903		
501		35.99	16.22		0.584	556.51	23.957	43.048		
503		18.83	107.89		2.031	575.33	25.988	45.170		
504		144.65	10.9		1.577	719.99	27.565	38.285		
505		14.60	23.86		0.348	734.58	27.913	37.998		
506		26.89	8.37		0.225	761.48	28.138	36.952		
P5			29.56			761.48	28.138	36.952	883.75	26.124
603		13.57	3.03		0.041	775.05	28.179	36.358		
612		109.26	16.24		1.774	884.31	29.953	33.872		

P6	27.29	884.31	29.953	33.872	1023.07	27.920

[a] Minus value means the amount that flow out from the mainstream. **Column A:** anthropogenic water discharges (m^3/min); **Column B:** water discharge in each Tributary (m^3/min); **Column C:** Zn concentrations in water samples analyzed by ICP-MS (μg/kg); **Column D:** Zn concentrations in human emissions (μg/kg); **Column E:** heavy metal outflows (g/min) calculated by B * C/1000; **Column F:** accumulated water discharge (m^3/min), the sum of all the water discharges upstream, calculated as F1 = A1 + B1, F2 = F1 + (A2 + B2), F3 = F2 + (A3 + B3); **Column G:** accumulated Heavy metal flows (g/min), calculated as G1 = A1 * D1+B1 * C1, G2 = G1+(A2 * D2+B2 * C2), G3 = G2+(A3 * D3+B3 * D3); **Column H:** estimated Zn concentration in mainstream (μg/kg), calculated by G/F * 1000; **Column I:** actually Measured water discharge at monitoring points(m^3/min); **Column J:** observed heavy metal flows at monitoring points (g/min), calculated by C * I/1000.

Table S2. Polygons' information.

Section	Polygon Name	Attribution	Polygon Area (km^2)	Rice Field Area (ha)[a]
	101	tributary	2.34	0.00
–P1	102	tributary	1.64	0.00
	P1	monitoring point		
	201	tributary	2.23	0.00
	202	mainstream	0.43	0.00
	203	tributary	1.18	0.00
P1–P2	204	mainstream	4.70	20.52
	205	tributary	8.91	9.22
	206	mainstream	0.55	24.00
	P2	monitoring point		
	301	tributary	9.67	19.43
	302	tributary	27.76	97.00
P2–P3	303	tributary	7.01	22.62
	304	tributary	22.03	43.54
	305	mainstream	3.16	56.09
	P3	monitoring point		
	401	tributary	4.76	10.33
P3–P4	402	mainstream	1.85	0.00
	P4	monitoring point		
	501	tributary	5.62	0.00
	502	mainstream	4.71	0.00
	503	tributary	2.94	0.00
P4–P5	504	tributary	22.59	37.57
	505	tributary	2.28	0.00
	506	tributary	4.20	1.10
	P5	monitoring point		
P5–P6	603	tributary	3.67	10.00

612	tributary	29.55	152.45
604	mainstream	4.46	0.00
P6	monitoring point		

[a] The data about paddy field area was extracted from land use/land cover information in Akita Prefecture.

Table S3. Calculations about Cu flows into mainstream and the accumulation effects in high-water season (May 2015).

	Anthropogenic Water Flows (m³/min)	Tributary Water Discharges (m³/min)	Cu Concentration Analyzed by ICP-MS(μg/kg)	Cu Concentration in Human Emissions (μg/kg)	Heavy Metal Outflows (g/min)	Accumulated Water Discharge (m³/min)	Accumulated Heavy Metal Flows (g/min)	Estimated Cu Concentration in Mainstream (μg/kg)	Actually Measured Water Discharge (m³/min)	Observed Heavy Metal Flows at Monitoring Points (g/min)
	A	B	C	D	E	F	G	H	I	J
101		18.04	0.27		0.005	18.04	0.005	0.270		
102		12.64	0.42		0.005	30.68	0.010	0.332		
P1			0.51			30.68	0.010	0.332	30.68	0.016
201		11.68	2.43		0.028	42.36	0.039	0.910		
203		6.18	0.65		0.004	48.54	0.041	0.835		
Furutobe Mine	0.23			8.603	0.002	48.77	0.043	0.872		
205		46.67	4.05		0.189	95.45	0.232	2.426		
Intake to Kosaka Refinery	−4.00 [a]				−0.010 [a]	91.45	0.222	2.426		
P2			2.3			91.45	0.222	2.426	121.20	0.279
301		62.37	0.4		0.025	153.81	0.247	1.604		
302		179.04	0.74		0.132	332.86	0.379	1.139		
303		45.21	0.68		0.031	378.07	0.410	1.085		
304		142.09	0.89		0.126	520.15	0.536	1.031		
P3			1.27			520.15	0.536	1.031	569.32	0.723
Intake to Irrigation Channel	-53.50 [a]				−0.055 [a]	466.65	0.481	1.031		
401		32.87	2.55		0.084	499.52	0.565	1.131		
P4			1.55			499.52	0.565	1.131	562.50	0.872
Kosaka refinery	21.00			48.02	1.008	520.52	1.574	3.023		
501		35.99	1.21		0.044	556.51	1.617	2.906		
503		18.83	9.37		0.176	575.33	1.793	3.117		

504		144.65	1.67		0.242	719.99	2.035	2.827		
505		14.60	1.1		0.016	734.58	2.051	2.792		
506		26.89	0.58		0.016	761.48	2.067	2.714		
P5			3.68			761.48	2.067	2.714	883.75	3.252
603		13.57	0.29		0.004	775.05	2.071	2.672		
612		109.26	1.88		0.205	884.31	2.276	2.574		
P6			4.13			884.31	2.276	2.574	1023.07	4.225

[a] Minus value means the amount that flow out from the mainstream. **Column A:** anthropogenic water discharges (m^3/min); **Column B:** water discharge in each Tributary (m^3/min); **Column C:** Cu concentrations in water samples analyzed by ICP-MS (μg/kg); **Column D:** Cu concentrations in human emissions (μg/kg); **Column E:** heavy metal outflows (g/min) calculated by B * C/1000; **Column F:** accumulated water discharge (m^3/min), the sum of all the water discharges upstream, calculated as F1 = A1 + B1, F2 = F1 + (A2 + B2), F3 = F2 + (A3 + B3); **Column G:** accumulated Heavy metal flows (g/min), calculated as G1 = A1 * D1+B1 * C1, G2 = G1+(A2 * D2+B2 * C2), G3 = G2+(A3 * D3+B3 * D3); **Column H:** estimated Cu concentration in mainstream (μg/kg), calculated by G/F * 1000; **Column I:** actually Measured water discharge at monitoring points(m^3/min); **Column J:** observed heavy metal flows at monitoring points (g/min), calculated by C * I/1000.

Table S4. Calculations about Pb flows into mainstream and the accumulation effects in high-water season (May 2015).

	**Anthropogenic Water flows (m^3/min)**	**Tributary Water Discharges (m^3/min)**	**Pb Concentration Analyzed by ICP-MS (μg/kg)**	**Pb Concentration in Human Emissions (μg/kg)**	**Heavy Metal Outflows (mg/min)**	**Accumulated Water Discharge (m^3/min)**	**Accumulated Heavy Metal Flows (mg/min)**	**Estimated Pb Concentration in Mainstream (μg/kg)**	**Actually Measured Water Discharge (m^3/min)**	**Observed Heavy Metal Flows at Monitoring Points (mg/min)**
	A	B	C	D	E	F	G	H	I	J
101		18.04	0.05		0.902	18.04	0.902	0.050		
102		12.64	0.02		0.253	30.68	1.155	0.038		
P1			0.03			30.68	1.155	0.038	30.68	0.920
201		11.68	0.04		0.467	42.36	1.622	0.038		
203		6.18	0.04		0.247	48.54	1.710	0.035		
Furutobe Mine	0.23			0.383	0.088	48.77	1.798	0.037		
205		46.67	0.04		1.867	95.45	3.665	0.038		
Intake to Kosaka Refinery	-4.00 [a]				-0.154 [a]	91.45	3.512	0.038		
P2			0.09			91.45	3.512	0.038	121.20	10.908
301		62.37	0.04		2.495	153.81	6.006	0.039		
302		179.04	0.03		5.371	332.86	11.377	0.034		
303		45.21	0.04		1.808	378.07	13.186	0.035		

304		142.09	0.16		22.734	520.15	35.920	0.069		
P3			0.14			520.15	35.920	0.069	569.32	79.705
Intake to Irrigation Channel	−53.50 [a]				−3.694 [a]	466.65	32.225	0.069		
401		32.87	0.45		14.791	499.52	47.016	0.094		
P4			0.23			499.52	47.016	0.094	562.50	129.375
Kosaka Refinery	21.00			2.356	49.476	520.52	96.492	0.185		
501		35.99	0.09		3.239	556.51	99.731	0.179		
503		18.83	0.24		4.518	575.33	104.249	0.181		
504		144.65	0.18		26.037	719.99	130.286	0.181		
505		14.60	0.17		2.482	734.58	132.768	0.181		
506		26.89	0.1		2.689	761.48	135.458	0.178		
P5			0.16			761.48	135.458	0.178	883.75	141.400
603		13.57	0.07		0.950	775.05	136.408	0.176		
612		109.26	0.16		17.482	884.31	153.889	0.174		
P6			0.14			884.31	153.889	0.174	1023.07	143.230

[a] Minus value means the amount that flow out from the mainstream. **Column A:** anthropogenic water discharges (m^3/min); **Column B:** water discharge in each Tributary (m^3/min); **Column C:** Pb concentrations in water samples analyzed by ICP-MS (μg/kg); **Column D:** Pb concentrations in human emissions (μg/kg); **Column E:** heavy metal outflows (g/min) calculated by B * C/1000; **Column F:** accumulated water discharge (m^3/min), the sum of all the water discharges upstream, calculated as F1 = A1 + B1, F2 = F1 + (A2 + B2), F3 = F2 + (A3 + B3); **Column G:** accumulated Heavy metal flows (g/min), calculated as G1 = A1 * D1+B1 * C1, G2 = G1+(A2 * D2+B2 * C2), G3 = G2+(A3 * D3+B3 * D3); **Column H:** estimated Pb concentration in mainstream (μg/kg), calculated by G/F * 1000; **Column I:** actually Measured water discharge at monitoring points(m^3/min); **Column J:** observed heavy metal flows at monitoring points (g/min), calculated by C * I/1000.

Table S5. Calculations about As flows into mainstream and the accumulation effects in high-water season (May 2015).

	Anthropogenic Water Flows (m^3/min)	Tributary Water Discharges (m^3/min)	As Concentration Analyzed by ICP-MS (μg/kg)	As Concentration in Human Emissions (mg/kg)	Heavy Metal Outflows (mg/min)	Accumulated Water Discharge (m^3/min)	Accumulated Heavy Metal Flows (mg/min)	Estimated As Concentration in Mainstream (μg/kg)	Actually Measured Water Discharge (m^3/min)	Observed Heavy Metal Flows at Monitoring Points (mg/min)
	A	B	C	D	E	F	G	H	I	J
101		18.04	0.13		2.345	18.04	2.345	0.130		
102		12.64	0.11		1.391	30.68	3.736	0.122		
P1			0.11			30.68	3.736	0.122	30.68	3.375
201		11.68	0.19		2.219	42.36	5.955	0.141		
203		6.18	0.17		1.051	48.54	6.218	0.128		

Furutobe Mine	0.23			1.144	0.263	48.77	6.481	0.133		
205		46.67	0.38		17.736	95.45	24.217	0.254		
Intake to Kosaka Refinery	−4.00 [a]				−1.015 [a]	91.45	23.202	0.254		
P2			0.31			91.45	23.202	0.254	121.20	37.572
301		62.37	0.68		42.410	153.81	65.612	0.427		
302		179.04	0.24		42.970	332.86	108.582	0.326		
303		45.21	0.27		12.207	378.07	120.790	0.319		
304		142.09	0.46		65.359	520.15	186.149	0.358		
P3			0.5			520.15	186.149	0.358	569.32	284.660
Intake to Irrigation Channel	−53.50 [a]				−19.146 [a]	466.65	167.003	0.358		
401		32.87	0.23		7.560	499.52	174.563	0.349		
P4			0.37			499.52	174.563	0.349	562.50	208.125
Kosaka Refinery	21.00			2.537	53.277	520.52	227.840	0.438		
501		35.99	0.39		14.035	556.51	241.874	0.435		
503		18.83	0.23		4.330	575.33	246.204	0.428		
504		144.65	0.62		89.683	719.99	335.888	0.467		
505		14.60	0.45		6.570	734.58	342.458	0.466		
506		26.89	0.27		7.261	761.48	349.719	0.459		
P5			0.64			761.48	349.719	0.459	883.75	565.600
603		13.57	0.18		2.443	775.05	352.161	0.454		
612		109.26	0.9		98.334	884.31	450.495	0.509		
P6			0.69			884.31	450.495	0.509	1023.07	705.918

[a] Minus value means the amount that flow out from the mainstream. **Column A:** anthropogenic water discharges (m^3/min); **Column B:** water discharge in each Tributary (m^3/min); **Column C:** As concentrations in water samples analyzed by ICP-MS (μg/kg); **Column D:** As concentrations in human emissions (μg/kg); **Column E:** heavy metal outflows (g/min) calculated by B * C/1000; **Column F:** accumulated water discharge (m^3/min), the sum of all the water discharges upstream, calculated as F1 = A1 + B1, F2 = F1 + (A2 + B2), F3 = F2 + (A3 + B3); **Column G:** accumulated Heavy metal flows (g/min), calculated as G1 = A1 * D1+B1 * C1, G2 = G1 + (A2 * D2+B2 * C2), G3 = G2+(A3 * D3+B3 * D3); **Column H:** estimated As concentration in mainstream (μg/kg), calculated by G/F * 1000; **Column I:** actually Measured water discharge at monitoring points(m^3/min); **Column J:** observed heavy metal flows at monitoring points (g/min), calculated by C * I/1000.

Table S6. Calculations about Zn flows into mainstream and the accumulation effects in low-water season (Oct 2015).

	Anthropogenic Water Flows (m^3/min)	Tributary Water Discharges (m^3/min)	Zn Concentration Analyzed by ICP-MS (μg/kg)	Zn Concentration in Human Emissions (μg/kg)	Heavy Metal Outflows (g/min)	Accumulated Water Discharge (m^3/min)	Accumulated Heavy Metal Flows (g/min)	Estimated Zn Concentration in Mainstream (μg/kg)	Actually Measured Water Discharge (m^3/min)	Observed Heavy Metal Flows at Monitoring Points (g/min)
	A	B	C	D	E	F	G	H	I	J
101		3.65	3.45		0.013	3.65	0.013	3.450		
102		2.56	1.56		0.004	6.21	0.017	2.671		
P1			2.72			6.21	0.017	2.671	6.21	0.017
201		2.78	27.6		0.077	8.99	0.093	10.378		
203		1.47	1.49		0.002	10.46	0.114	10.936		
Furutobe Mine	0.23			91.741	0.021	10.69	0.135	12.675		
205		11.10	41.16		0.457	21.79	0.592	27.188		
Intake to Kosaka Refinery	−4.00 [a]				−0.109 [a]	17.79	0.484	27.188		
P2			25.08			17.79	0.484	27.188	24.87	0.624
301		18.10	1.1		0.020	35.89	0.504	14.031		
302		51.96	1.6		0.083	87.86	0.587	6.679		
303		13.12	8.32		0.109	100.98	0.696	6.892		
304		41.24	2.31		0.095	142.22	0.791	5.563		
P3			28.54			142.22	0.791	5.563	154.93	4.422
Intake to Irrigation Channel	−53.50 [a]				−0.298 [a]	88.72	0.494	5.563		
401		9.44	419		3.953	98.15	4.447	45.307		
P4			33.63			98.15	4.447	45.307	114.83	3.862
Kosaka Refinery	21.00			253.678	5.327	119.15	9.774	82.031		
501		9.15	33.5		0.306	128.30	10.081	78.572		
503		4.78	89.96		0.430	133.08	10.511	78.981		
504		36.76	13.53		0.497	169.85	11.008	64.814		
505		3.71	17.95		0.067	173.56	11.075	63.812		
506		6.84	5.04		0.034	180.39	11.110	61.586		
P5			44.73			180.39	11.110	61.586	233.82	10.459
603		1.37	1.64		0.002	181.76	11.112	61.134		
612		11.03	12.19		0.135	192.80	11.246	58.332		
P6			41.25			192.80	11.246	58.332	247.89	10.225

[a] Minus value means the amount that flow out from the mainstream. **Column A:** anthropogenic water discharges (m^3/min); **Column B:** water discharge in each Tributary (m3/min); **Column C:** Zn concentrations in water samples analyzed by ICP-MS (μg/kg); **Column D:** Zn concentrations in human emissions (μg/kg);

Column E: heavy metal outflows (g/min) calculated by B * C/1000; **Column F:** accumulated water discharge (m3/min), the sum of all the water discharges upstream, calculated as F1 = A1 + B1, F2 = F1 + (A2 + B2), F3 = F2 + (A3 + B3); **Column G:** accumulated Heavy metal flows (g/min), calculated as G1 = A1 * D1 + B1 * C1, G2 = G1+(A2 * D2+B2 * C2), G3 = G2 + (A3 * D3+B3 * D3); **Column H:** estimated Zn concentration in mainstream (μg/kg), calculated by G/F * 1000; **Column I:** actually Measured water discharge at monitoring points(m^3/min); **Column J:** observed heavy metal flows at monitoring points (g/min), calculated by C * I/1000.

Table S7. Calculations about Cu flows into mainstream and the accumulation effects in low-water season (Oct 2015).

	**Anthropogenic Water Flows (m^3/min)**	**Tributary Water Discharges (m^3/min)**	**Cu Concentration Analyzed by ICP-MS(μg/kg)**	**Cu Concentration in Human Emissions (μg/kg)**	**Heavy Metal Outflows (g/min)**	**Accumulated Water Discharge (m^3/min)**	**Accumulated Heavy Metal Flows (g/min)**	**Estimated Cu Concentration in Mainstream (μg/kg)**	**Actually Measured Water Discharge (m^3/min)**	**Observed Heavy Metal Flows at Monitoring Points (g/min)**
	A	B	C	D	E	F	G	H	I	J
101		3.65	0.79		0.003	3.65	0.003	0.790		
102		2.56	0.82		0.002	6.21	0.005	0.802		
P1			0.74			6.21	0.005	0.802	6.21	0.005
201		2.78	3.44		0.010	8.99	0.015	1.618		
203		1.47	1.92		0.003	10.46	0.017	1.580		
Furutobe Mine	0.23			8.603	0.002	10.69	0.018	1.731		
205		11.10	3.91		0.043	21.79	0.062	2.841		
Intake to Kosaka Refinery	−4.00 [a]				−0.011 [a]	17.79	0.051	2.841		
P2			2.83			17.79	0.051	2.841	24.87	0.070
301		18.10	0.57		0.010	35.89	0.061	1.696		
302		51.96	0.81		0.042	87.86	0.103	1.172		
303		13.12	1.24		0.016	100.98	0.119	1.181		
304		41.24	0.91		0.038	142.22	0.157	1.102		
P3			3.74			142.22	0.157	1.102	154.93	0.579
Intake to Irrigation Channel	−53.50 [a]				−0.059 [a]	88.72	0.098	1.102		
401		9.44	3.34		0.032	98.15	0.129	1.317		
P4			3.47			98.15	0.129	1.317	114.83	0.398
Kosaka Refinery	21.00			48.02	1.008	119.15	1.138	9.548		

501		9.15	3.73		0.034	128.30	1.172	9.134		
503		4.78	7.96		0.038	133.08	1.210	9.091		
504		36.76	2.1		0.077	169.85	1.287	7.578		
505		3.71	1.42		0.005	173.56	1.292	7.446		
506		6.84	0.61		0.004	180.39	1.297	7.187		
P5			5.7			180.39	1.297	7.187	233.82	1.333
603		1.37	0.36		0.000	181.76	1.297	7.136		
612		11.03	2.26		0.025	192.80	1.322	6.857		
P6			5.42			192.80	1.322	6.857	247.89	1.344

[a] Minus value means the amount that flow out from the mainstream. **Column A:** anthropogenic water discharges (m^3/min); **Column B:** water discharge in each Tributary (m^3/min); **Column C:** Cu concentrations in water samples analyzed by ICP-MS (μg/kg); **Column D:** Cu concentrations in human emissions (μg/kg); **Column E:** heavy metal outflows (g/min) calculated by B*C/1000; **Column F:** accumulated water discharge (m^3/min), the sum of all the water discharges upstream, calculated as F1 = A1 + B1, F2 = F1 + (A2 + B2), F3 = F2 + (A3 + B3); **Column G:** accumulated Heavy metal flows (g/min), calculated as G1=A1 * D1+B1 * C1, G2 = G1+(A2 * D2+B2 * C2), G3 = G2+(A3 * D3+B3 * D3); **Column H:** estimated Cu concentration in mainstream (μg/kg), calculated by G/F * 1000; **Column I:** actually Measured water discharge at monitoring points(m^3/min); **Column J:** observed heavy metal flows at monitoring points (g/min), calculated by C * I/1000.

Table S8. Calculations about Pb flows into mainstream and the accumulation effects in low-water season (Oct 2015).

	**Anthropogenic Water Flows (m^3/min)**	**Tributary Water Discharges (m^3/min)**	**Pb Concentration Analyzed by ICP-MS (μg/kg)**	**Pb Concentration in Human Emissions (μg/kg)**	**Heavy Metal Outflows (mg/min)**	**Accumulated Water Discharge (m^3/min)**	**Accumulated Heavy Metal Flows (mg/min)**	**Estimated Pb Concentration in Mainstream (μg/kg)**	**Actually Measured Water Discharge (m^3/min)**	**Observed Heavy Metal Flows at Monitoring Points (mg/min)**
	A	B	C	D	E	F	G	H	I	J
101		3.65	0.13		0.475	3.65	0.475	0.130		
102		2.56	0.06		0.154	6.21	0.628	0.101		
P1			0.07			6.21	0.628	0.101	6.21	0.435
201		2.78	0.09		0.250	8.99	0.878	0.098		
203		1.47	0.07		0.103	10.46	0.966	0.092		
Furutobe Mine	0.23			0.383	0.088	10.69	1.054	0.099		
205		11.10	0.1		1.110	21.79	2.165	0.099		
Intake to Kosaka Refinery	−4.00 [a]				−0.397 [a]	17.79	1.767	0.099		
P2			0.15			17.79	1.767	0.099	24.87	3.731
301		18.10	0.05		0.905	35.89	2.672	0.074		

302		51.96	0.12		6.236	87.86	8.908	0.101		
303		13.12	0.06		0.787	100.98	9.695	0.096		
304		41.24	0.41		16.908	142.22	26.603	0.187		
P3			0.27			142.22	26.603	0.187	154.93	41.831
Intake to Irrigation Channel	−53.50 [a]				−10.008 [a]	88.72	16.595	0.187		
401		9.44	1.53		14.436	98.15	31.032	0.316		
P4			0.3			98.15	31.032	0.316	114.83	34.449
Kosaka Refinery	21.00			2.356	49.476	119.15	80.508	0.676		
501		9.15	0.37		3.384	128.30	83.892	0.654		
503		4.78	0.19		0.909	133.08	84.801	0.637		
504		36.76	0.19		6.985	169.85	91.786	0.540		
505		3.71	0.13		0.482	173.56	92.268	0.532		
506		6.84	0.16		1.094	180.39	93.362	0.518		
P5			0.37			180.39	93.362	0.518	233.82	86.513
603		1.37	0.04		0.055	181.76	93.417	0.514		
612		11.03	0.22		2.428	192.80	95.844	0.497		
P6			0.26			192.80	95.844	0.497	247.89	64.451

[a] Minus value means the amount that flow out from the mainstream. **Column A:** anthropogenic water discharges (m^3/min); **Column B:** water discharge in each Tributary (m^3/min); **Column C:** Pb concentrations in water samples analyzed by ICP-MS (μg/kg); **Column D:** Pb concentrations in human emissions (μg/kg); **Column E:** heavy metal outflows (g/min) calculated by B * C/1000; **Column F:** accumulated water discharge (m^3/min), the sum of all the water discharges upstream, calculated as F1 = A1 + B1, F2 = F1 + (A2 + B2), F3 = F2 + (A3 + B3); **Column G:** accumulated Heavy metal flows (g/min), calculated as G1 = A1 * D1+B1 * C1, G2 = G1+(A2 * D2+B2 * C2), G3 = G2+(A3 * D3+B3 * D3); **Column H:** estimated Pb concentration in mainstream (μg/kg), calculated by G/F * 1000; **Column I:** actually Measured water discharge at monitoring points(m^3/min); **Column J:** observed heavy metal flows at monitoring points (g/min), calculated by C * I/1000.

Table S9. Calculations about As flows into mainstream and the accumulation effects in low-water season (Oct 2015).

	**Anthropogenic Water Flows (m^3/min)**	**Tributary Water Discharges (m^3/min)**	**As Concentration Analyzed by ICP-MS (μg/kg)**	**As Concentration in Human Emissions (μg/kg)**	**Heavy Metal Outflows (mg/min)**	**Accumulated Water Discharge (m^3/min)**	**Accumulated Heavy Metal Flows (mg/min)**	**Estimated As Concentration in Mainstream (μg/kg)**	**Actually Measured Water Discharge (m^3/min)**	**Observed Heavy Metal Flows at Monitoring Points (mg/min)**
	A	B	C	D	E	F	G	H	I	J
101		3.65	0.14		0.511	3.65	0.511	0.140		
102		2.56	0.2		0.512	6.21	1.023	0.165		

P1			0.14			6.21	1.023	0.165	6.21	0.869
201		2.78	0.23		0.639	8.99	1.662	0.185		
203		1.47	0.16		0.235	10.46	1.925	0.184		
Furutobe Mine	0.23			1.144	0.263	10.69	2.188	0.205		
205		11.10	0.53		5.885	21.79	8.073	0.370		
Intake to Kosaka Refinery	−4.00 [a]				−1.482 [a]	17.79	6.591	0.370		
P2			0.35			17.79	6.591	0.370	24.87	8.705
301		18.10	0.7		12.671	35.89	19.262	0.537		
302		51.96	0.35		18.187	87.86	37.449	0.426		
303		13.12	0.45		5.905	100.98	43.354	0.429		
304		41.24	1.32		54.434	142.22	97.789	0.688		
P3			0.62			142.22	97.789	0.688	154.93	96.057
Intake to Irrigation Channel	−53.50 [a]				−36.786 [a]	88.72	61.002	0.688		
401		9.44	0.2		1.887	98.15	62.889	0.641		
P4			0.24			98.15	62.889	0.641	114.83	27.559
Kosaka Refinery	21.00			2.537	53.277	119.15	116.166	0.975		
501		9.15	0.64		5.853	128.30	122.020	0.951		
503		4.78	0.64		3.062	133.08	125.082	0.940		
504		36.76	0.35		12.867	169.85	137.949	0.812		
505		3.71	0.31		1.150	173.56	139.099	0.801		
506		6.84	0.8		5.468	180.39	144.567	0.801		
P5			1.14			180.39	144.567	0.801	233.82	266.555
603		1.37	0.17		0.233	181.76	144.800	0.797		
612		11.03	1.2		13.241	192.80	158.041	0.820		
P6			1.11			192.80	158.041	0.820	247.89	275.158

[a] Minus value means the amount that flow out from the mainstream. **Column A:** anthropogenic water discharges (m^3/min); **Column B:** water discharge in each Tributary (m^3/min); **Column C:** As concentrations in water samples analyzed by ICP-MS (μg/kg); **Column D:** As concentrations in human emissions (μg/kg); **Column E:** heavy metal outflows (g/min) calculated by B * C/1000; **Column F:** accumulated water discharge (m^3/min), the sum of all the water discharges upstream, calculated as F1 = A1 + B1, F2 = F1 + (A2 + B2), F3 = F2 + (A3 + B3); **Column G:** accumulated Heavy metal flows (g/min), calculated as G1 = A1 * D1+B1 * C1, G2 = G1+(A2 * D2+B2 * C2), G3 = G2+(A3 * D3+B3 * D3); **Column H:** estimated As concentration in mainstream (μg/kg), calculated by G/F * 1000; **Column I:** actually Measured water discharge at monitoring points(m^3/min); **Column J:** observed heavy metal flows at monitoring points (g/min), calculated by C * I/1000.
